# Hot Deformation Behavior of 4130 High-Strength Steel

**DOI:** 10.3390/ma15217817

**Published:** 2022-11-05

**Authors:** Aneta Łukaszek-Sołek, Tomasz Śleboda, Łukasz Lisiecki, Janusz Krawczyk

**Affiliations:** Faculty of Metals Engineering and Industrial Computer Science, AGH University of Science and Technology, Av. Mickiewicza 30, 30-059 Krakow, Poland

**Keywords:** thermomechanical processing, steel, processing maps, microstructure, forging

## Abstract

Hot deformation behavior of 4130 steel and optimization of its processing parameters are presented in this paper. Compression tests were performed at temperatures ranging from 800 to 1200 °C and at the strain rates in the range from 0.01 to 100 s^−1^. A comprehensive analysis of the material behavior at different temperature and strain-rate ranges was performed taking into account various criteria of stability and instability of the material flow under various thermomechanical conditions. The flow–stress curves obtained during compression tests, as well as the processing maps elaborated on the basis of various flow–stability criteria, are discussed. Processing parameters developed according to the Prasad’s and Murty’s criteria are recommended for designing the technology of forging of the investigated steel. Such parameters ensure the homogeneity and stability of the material flow in a forged part, what was confirmed by successful forging of 4130 steel in industrial conditions. The processing map developed according to Gegel’s approach, as compared to the processing maps obtained in accordance with the Prasad’s and Murty’s criteria, should be treated as general support for determining the thermomechanical processing parameters.

## 1. Introduction

The control of hot deformation behavior and the properly chosen processing parameters are essential for successful design of the technology of the production of high-strength steel parts [1,2,3,4]. Processing maps and dynamic material modeling [5,6,7] enable selecting appropriate parameters of thermomechanical processing of such steels and, as a result, obtaining good quality products having profitable mechanical properties and uniform microstructure. Much research in this area has been carried out in relation to aluminum alloys [8,9], titanium alloys [10,11], and nickel alloys [12,13]. In the design of structural parts widely used in the automotive and railroad industries, high-strength steels are becoming increasingly important [14,15].

Using the basic principles of continuum mechanics of large plastic flow and also modeling of physical systems and irreversible thermodynamics, a methodology called the dynamic material model was developed [16]. The workpiece is treated as the dissipator of power—the unit power (*P*) absorbed by the material during processing. It can be expressed as [17,18]:

(1) $$P=\sigma \cdot \dot{\varepsilon }=G+J=\int_{0}^{\dot{\varepsilon }}\sigma_{p}d\dot{\varepsilon }+\int_{0}^{\sigma }\dot{\varepsilon }d\sigma$$

where $\dot{\varepsilon }$ = the strain rate, s^−1^, *σ* = the flow stress, MPa, *J* = dissipator co-content, *G =* dissipator content. 

The *J* co-content, associated with the power dissipation dynamic processes ongoing in the material being processed, can be expressed as follows [18]:

(2) $$J=\int_{0}^{\sigma }\dot{\varepsilon }d\sigma =\frac{\sigma \cdot \dot{\varepsilon }\cdot m}{m+1}$$

where *m* is the strain-rate-sensitivity parameter. 

The relationship between the heat generation (*G*) and the microstructural changes (*J*) is described by the strain-rate-sensitivity parameter (m) and can be calculated from the formula [17]:

(3) $$m={\left(\frac{\partial J}{\partial G}\right)}_{T,\;\varepsilon }={\left(\frac{\partial \log\sigma }{\partial \log\dot{\varepsilon }}\right)}_{T,\;\varepsilon }$$

where *ε* = the constant true strain value, *T* = the temperature, °C.

Flow–stress data are used to derive the workability parameters by applying various criteria and developing processing maps from them. The processing maps as a function of temperature and log (strain rate) show the process conditions for stable and unstable hot deformation [19,20,21,22]. For the determination of the stability domains and the areas of flow instability based on the dynamic modeling of the material behavior in hot deformation processes, various criteria have been developed. The criteria for stability and instability of a material under different thermomechanical conditions make it possible to conduct research on the behavior of metallic materials in hot deformation processes. Such studies should also consider the comparison of existing criteria [23,24,25]. Nevertheless, the responsibility for selecting and evaluating the correctness of a given theory in modeling hot deformation behavior is within the scope of the designer or researcher responsibility.

In the first criterion (Prasad’s approach), the efficiency of power dissipation-η, reflecting the material microstructure evolution, is determined by the following formula [7]:

(4) $$\eta =\frac{J}{J_{\max}}=\frac{2m}{m+1}$$

This criterion can be expressed as [7,26,27]:

(5) $$\xi_{1}=\frac{\partial \log\left(\frac{m}{m+1}\right)}{\partial \log\dot{\varepsilon }}+m\leq 0$$

It is used to identify instabilities in the material flow during hot working.

In the second criterion, according to Murty’s approach, the expression of the power dissipation efficiency *η* is defined by the use of the *J*/*Jmax* ratio, and provided by [28,29]:

(6) $$\eta =\frac{J}{J_{max}}=2\left(1-\frac{1}{\sigma \dot{\varepsilon }}\int_{0}^{\dot{\varepsilon }}\sigma \;d\dot{\varepsilon }\right)$$

The Murty’s instability parameter can be calculated as [28,29,30]:

(7) $$\xi_{2}=\frac{2m}{\eta }-1<0$$

Applying the second law of thermodynamics, the extremal principles of irreversible thermodynamics as applied to large plastic flow and the stability theory, Gegel [23,29] constructed two Liapunov functions that applied the efficiency of the power dissipation *η* and the temperature sensitivity s with the independence variable as $\log\dot{\varepsilon }$:

(8) $$V_{1}=\eta \left(\log\dot{\varepsilon }\right)$$

(9) $$V_{2}=s\left(\log\dot{\varepsilon }\right)$$

The temperature-sensitivity parameter of the flow stress can be calculated from the equation [29]:

(10) $$s=\frac{1}{T}\cdot \frac{\partial \ln\sigma }{\partial \left(\frac{1}{T}\right)}$$

The parameters η and *s* are determined as the strain rate and temperature function.

In the third criterion, according to Gegel’s approach, the criteria for ensuring the stable material flow could be given as:

(11) 0<m≤1

(12) $$\eta_{criterion}=\frac{\partial \eta }{\partial \left(\ln\dot{\varepsilon }\right)}<0$$

(13) s≥1

(14) $$s_{criterion}=\frac{\partial s}{\partial \left(\ln\dot{\varepsilon }\right)}<0$$

The Gegel’s instability parameters are:

(15) $$\xi_{3}=m<0$$

(16) $$\xi_{4}=-\frac{\partial \eta }{\partial \left(\ln\dot{\varepsilon }\right)}<0$$

(17) $$\xi_{5}=s-1<0$$

(18) $$\xi_{6}=-\frac{\partial s}{\partial \left(\ln\dot{\varepsilon }\right)}<0$$

Considering Gegel’s criterion, the strain-rate-sensitivity parameter m can be related to the mechanical stability and the temperature-sensitivity parameter s to the material or structural stability [23]. In order to ensure stable material flow, it is important that the stress increases with the strain rate (and the curves have a convex shape), and the flow stress should decrease with respect to the temperature [28]. According to the second law of thermodynamics, if the temperature-sensitivity parameter s is greater than 1, then the stable material flow will be ensured [29]. In order to gain a power dissipation map, the stability parameters should be presented as a contour map taking into account temperature and log (strain rate). With such a map, it is possible to observe the proportion of power dissipated by the microstructure as a function of strain rate, strain, and temperature. It is assumed that the higher the value of the power dissipation efficiency (30–60%), the higher the probability of dynamic recrystallization (DRX), while at the lower values of the power dissipation efficiency (20–30%) DRV-related processes can be expected [12]. It happens that high values of the parameter η do not always correspond to domains that are safe for the workability of the material, and therefore the criterion for the occurrence of flow instability (η*_i_*) must also be taken into account. In the result, changes in the instability parameter η*_i_* as a function of strain rate and temperature enable instability maps to be plotted. The areas where η*_i_* < 0 correspond to microstructural instabilities in the material. In these areas, defects can be expected in the form of void formation, wedge cracking, intercrystalline fracture and other types of fracture processes, as well as adiabatic shear bands, Lüder bands, kink bands and mechanical twinning [21,31,32]. When designing the forming technology for a specific material, the information gained from the processing map is very useful. The process windows describe the most favorable forming parameters (in terms of temperature and strain rate) and also allow the control of the microstructure. The range of strain and strain rates under which the processing tests are conducted are also of great importance. Bulk metal forming processes are characterized by the wide range of deformation velocity and the corresponding wide range of the strain rates. In the case of structural parts with complex geometries, it seems worthwhile to undertake the studies comparing the accuracy of the criteria for identifying areas of the flow instability on the processing maps obtained according to Prasad’s, Murty’s, and Gegel’s criteria for the different strains and the highest possible strain rates. An interesting approach to analyze the deformation behaviour and microstructure evolution in isothermal compression of 300M steel was presented by Sun et al. [33]. The development of processing maps coupled with grain size was proposed using the physics-based visco-plastic constitutive equations and adopting a dynamic material model. For the different strains, the efficiency of the power dissipation and the instability parameters coupled with the material grain size were calculated. It was found that, in the case of isothermal compression of 300M steel, the grain size-coupled processing maps differ from the traditional processing maps in terms of Prasad’s theory, and that, in parallel with the microstructure observations, they can more accurately describe the deformation behavior of the material at elevated temperatures. Zhou et al. [34] studied the hot forging behavior of 25CrMo4 steel and developed a constitutive model. Moreover, the optimum parameters for the forging process were determined from the throughput maps and, using a kinetic model, the effect of strain on the efficiency of the power dissipation was discussed. However, the study was conducted for a true strain of 0.7 and a maximum strain rate of 10 s^−1^. Usually, processing maps are produced for only one, and a small, strain value. Only few works present processing maps developed for strains of, for example, 0.8 and higher [35,36,37,38]. For example, Liu et al. [39] studied the hot deformation behavior of 25CrMo4 steel based on the processing maps but only for the strain of 0.8 and strain rate of 1 s^−1^. Such a low strain rate does not correspond to the strain rates commonly used in industrial conditions in the majority of forging machines. In the majority of papers, the highest strain rate for which research is conducted is 10 s^–1^ [34,40], sometimes 50 s^–1^ [41], and infrequently 80 s^–1^ [25]. In turn, the strain rate of 100 s^–1^ was taken under consideration, for example, by Sescharyulu et al. [42], but only for the true strain of 0.5, Sui et al. [43] for the true strain of 0.7, Łukaszek-Sołek et al. [35,36] for the true strain of 0.9, and by Pu et al. [44] for the strain of 1. Few works deal with the comparison of different criteria and the development of comprehensive processing maps. For the high-Mn TWIP austenitic steel [37] instability maps based on different stability and instability criteria (i.e., Gegel’s and Alexander-Malas’s stability criterion, Prasad’s, Murty’s and Babu’s instability criterion) were developed and compared. On the basis of the deformation maps based on microstructural observation, it was found that instability maps differed to some extent from each other and that the instability criteria were more suitable in terms of predicting the occurrence of flow instabilities, which manifest themselves as heterogeneous flow and localized deformation bands.

This paper presents a comprehensive analysis of the hot deformation behavior of the 4130 steel under test at different temperature ranges and strain rates up to 100 s^−1^. The flow–stress curves obtained from the compression tests were used to develop processing maps developed on the basis of different flow–stability criteria for different strains up to that equal to 1. As a result, the hot deformation behavior of the investigated steel for a wide range of strain rates corresponding to the characteristics of most machines used in industrial forging conditions and a wide range of strains is discussed and, furthermore, favorable hot working parameters were proposed and verified in an industrial forging test.

## 2. Experimental Procedure 

4130 steel was selected for the investigation. Table 1 presents the chemical composition of the investigated steel. 

The investigated material was supplied by a commercial manufacturer (FRY Steel Company, Chicago, USA) in a form of a Ø50 bar in a heat-treated state. All test samples were cut out from a 4130-steel rod, in the direction parallel to the rod axis at a distance of approx. 2/3 of the radius from the center of the rod cross-section. The samples having 12 mm in height and 10 mm in diameter were machined by electrical discharge machining (EDM) and polished before compression tests. A compression testing was then performed on the Gleeble 3800 thermomechanical simulator (Dynamic Systems Inc., Poestenkill, NY, USA) until a total true strain was 1. The isothermal tests were carried out in an argon atmosphere at the temperature range from 800 to 1200 °C, and at the strain-rate range from 0.01–100 s^−1^. A graphite foil was used as a lubricant to minimize the friction between the samples and anvils. The samples were homogenized in 10 s prior to deformation and the resistance heating rate of 2.5 °C/s was applied. After the compression test, the specimens were cooled with compressed air (10 °C/s) and machined along the axial direction to observe the microstructure. Microstructural investigations were performed using an Axiovert 200MAT optical microscope, manufactured by the Zeiss company (Oberkochen, Germany), and also using a scanning microscope, FEI VERSA 3D (FEI—ThermoFisher Scientific, Waltham, MA, USA),, for obtaining images with the application of the BSE technique (the detector of reversely dissipated electrons). Analytical modeling was performed using Matlab software (version R2022a, MathWorks, Natick, MA, USA) in order to calculate the workability parameters such as the strain-rate-sensitivity parameter m, the efficiency of power dissipation, and etc. The various parameters needed for designing processing maps were calculated based on the flow–stress data. A cubic spline fit for the test data was applied to generate the greater number of data points. The Surfer software (version 23.3.202, Golden Software, LLC, Golden, USA) was used for generating the processing maps. Industrial drop forging tests were conducted for selected model forging made of the 4130 steels under the conditions determined for processing windows on developed processing maps.

## 3. Results and Discussion

**Figure 1:**
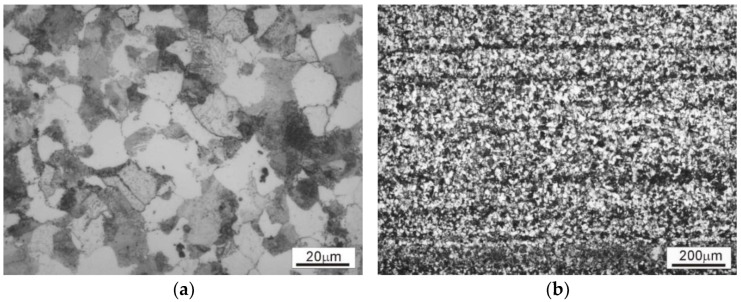
The microstructures of the 4130 steel in as-delivered condition: (**a**) transverse cross-section, (**b**) longitudinal cross-section.

The microstructure of the investigated steel in as-delivered (hot-rolled and normalized) condition is presented in Figure 1.

The microstructure of the starting material (annealed 4130 steel bar) consists of ferrite and pearlite (Figure 1a,b). In the longitudinal section, banding resulting from interdendritic microsegregation and subsequent hot forming can be observed (Figure 1b). The homogeneity of the material on the rod cross-section allowed for the preparation of samples with a similar initial microstructure for the plastometric tests. Additionally, the presence of weak banding allowed the assessment of the influence of deformation on the material flow and the influence of deformation conditions on the homogenization of the microstructure.

### 3.1. Flow Behaviour

**Figure 2:**
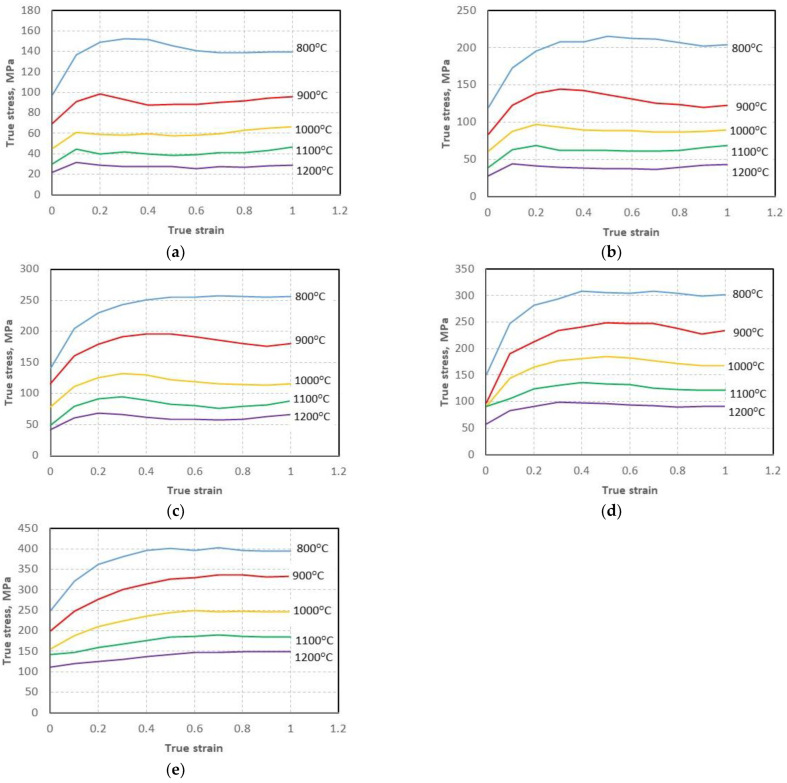
The true stress-true strain curves for the investigated 4130 steel deformed in compression at the true strain rate of 0.01 s^−1^ (**a**), 0.1 s^−1^ (**b**), 1 s^−1^ (**c**), 10 s^−1^ (**d**), 100 s^−1^ (**e**).

True stress–strain curves for the investigated steel deformed to a true strain of 1 at the temperature range from 800–1200 °C and strain-rate range from 0.01–100 s^−1^ are presented in Figure 2. 

The expected decrease in flow stress with increasing temperature and decreasing strain rate was observed, which signifies that, in the case of the investigated steel processing, the deformation temperature and strain rate have great effect on its hot deformation behavior. Stress rising to a peak followed by softening toward a steady state region signifies typical DRX behavior under higher temperatures (1000–1200 °C) regardless of strain rate. The flow–stress curves for the lower deformation temperatures (800 °C and 900 °C) indicate that, at those temperatures, the investigated material underwent recrystallization to a limited extent also regardless of the applied strain rate. 

### 3.2. Processing Maps

**Figure 3:**
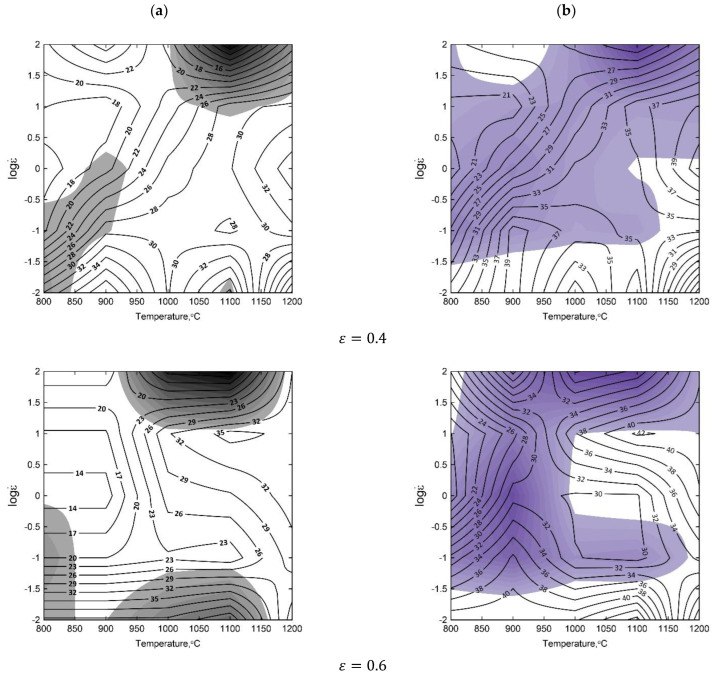

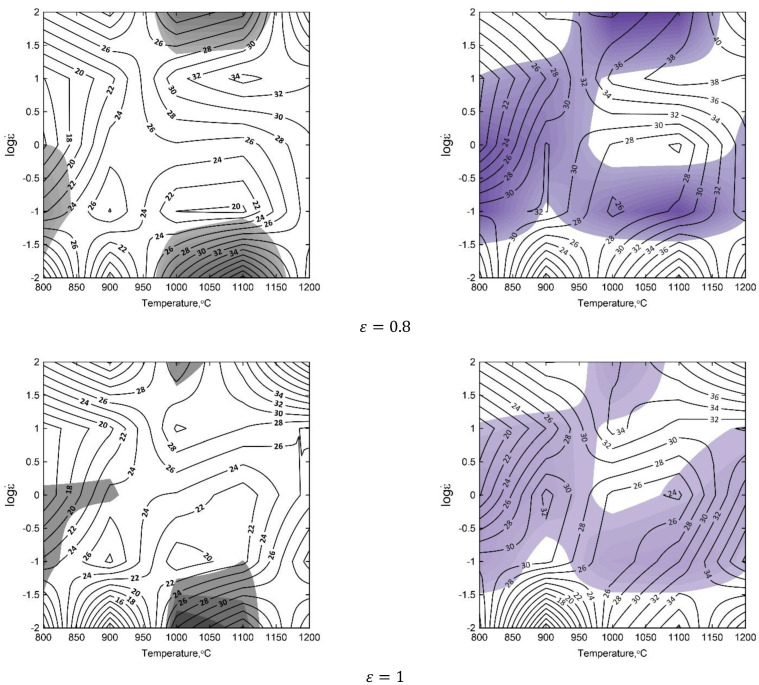
4130 steel processing maps based on the Prasad’s (**a**) and Murty’s (**b**) criterion for the true strain of: 0.4, 0.6, 0.8, 1.

Basing on the obtained flow–stress curves, the processing maps for various temperatures and for the true strains of ε = 0.4, 0.6, 0.8, and 1 were elaborated (Figure 3). The numbers on the contour lines signifies the percent efficiency of the power dissipation η. They are directly related to the process of microstructural evolution of the material, such as DRX, grain coarsening or DRV [45,46,47]. The areas of the flow instability characterize inhomogeneous deformation, which should be avoided when designing hot processing conditions. 

The areas of material flow instability shown on the processing maps were elaborated for a true strain of 1 (Figure 3). The preferred processing windows include the significant parameters of the process and design recommendations for the thermomechanical forging processes. In the processing maps drawn up upon the basis of Prasad’s criterion, three processing windows were differentiated and upon the basis of Murty’s criterion, there were four processing windows. Processing window 1 is characterized by the similar location on the processing map comparing Prasad’s with Murty’s approach within the range of *η*%, which represents the temperature and strain rate, and constitutes the contour of the dissipation efficiency parameter:

- $\eta_{P}=18–36\%$ in the range of temperatures from T=800–975 °C, and a strain rate of $\dot{\varepsilon }=12.5–100{\;s}^{–1}$. The isoclines of *η*%, retaining fairly uniform variability, have a regular oval shape and indicate an increasing workability of the material with an increasing strain rate.
- $\eta_{M}=18–32\%$ in the range of temperatures from T=800–950 °C, and a strain rate $\dot{\varepsilon }=12.5–100{\;s}^{–1}$. The isoclines of parameter *η* have a similar course, a small amplitude of changes, and they increase simultaneously with the temperature. The changing frequency of the course of the isoclines indicates the certain stability of material flow during deformation.

It may be recommended for the processes of small forgings with the application of the forging devices of the following types: horizontal forging machines, hydraulic screw presses, screw presses and double-action air-steam hammers.

The comparison of processing window 2 developed in accordance with Prasad’s and Murty’s criterion makes it possible to see their similarity (shape and parameters). It is situated within the contour of isoclines:

- $\eta_{P}=30–46\%$ in the range of temperatures from T=1075–1200 °C and strain rate of $\dot{\varepsilon }=15.8–100{\;s}^{–1}$.
- $\eta_{M}=32–44\%$ for T=1075–1200 °C and $\dot{\varepsilon }=15.8–100{\;s}^{–1}$.

The isoclines of the dissipation efficiency parameter *η*% have a very regular shape. That window reflects the optimum conditions for the material workability at the temperature of 1200 °C and a strain rate $100{\;s}^{–1}$. It may be recommended for conducting the conventional processes of die impression forging for a wide assortment of forgings with the application of the Maxi-type mechanical presses, screw presses and double-action air-steam hammers. For that group of forging devices, the deformation velocity converted into the strain rate fluctuates within a very wide range ($\dot{\varepsilon }=0.03-1400{\;s}^{-1}$).

Processing window 3 is characterized by the following parameters:

- $\eta_{P}=10–24\%$, $\;\dot{\varepsilon }=0.01\;–\;0.06{\;s}^{–1}$, and T=800–980 °C. The values of the *η*% isoclines may prove the uniformity of the material flow in the range of small strain rates, and possibly that window may be recommended for the purpose of hot forging small drop forgings with the application of a hydraulic press.
- $\eta_{M}=10\;–30\%$ in the range of temperatures from T=800–980 °C, and strain rate $\dot{\varepsilon }=0.01\;–\;0.06{\;s}^{–1}$. The course of the isoclines maintains a uniform growth tendency of the values of *η*% with the decrease in temperature down to 800 °C. The window is dedicated for hot forging small and very small, compact and elongated die forgings with the application of hydraulic presses, and the open die forgings in flat or shaped anvils.

Processing window 4 in accordance with Murty’s criterion includes the following parameters:

- $\eta_{M}=28–38\%$, T=1000–1200 °C and $\dot{\varepsilon }=0.01–0.03{\;s}^{–1}$. The window characterizes the optimal level of susceptibility to plastic deformation, reaching the maximum of the values of power dissipation amounting to 38%. The isoclines of the process efficiency *η* have a regular course and a small curvature in its shape, proving the uniform kinetics of the material plastic flow, and homogeneous deformation. The parameters of this window are appropriate for the forging of relatively small die forgings with the application of Maxi mechanical, hydraulic and hydraulic–mechanical presses.

The shape and size of the processing windows for ε=0.8 are similar to the windows for ε=1 and undergo the substantial differentiation of the significant parameters of the process. 

In the case of the true strain of 0.6, the areas of instability undergo extension and are not in agreement with processing window 3 obtained according to Prasad’s approach. The processing map generated for the true strain of 0.6, taking into consideration Murty’s approach, shows one very extensive area of instability, which is not in agreement with processing windows 1 and 2. Processing windows 3 and 4 change their parameters, because in this case the stability areas insignificantly change their size—they are flattened and elongated in their shape.

For the true strain of 0.4 on the processing map, there are only two processing windows according to Prasad’s approach: 1, and also 3 (but having slightly different position). Processing window 2 was excluded from consideration because of the increased area of instability. Taking into account the processing windows created on the processing map obtained according to Murty criterion, the position of windows 3 and 4 is not changed. The efficiency of power dissipation parameter *η*% has much lower values.

The processing parameters presented in processing window 2 are recommended for designing the technology of forging. They should ensure the homogeneity and stability of the material flow in a forged part. 

Table 2 shows a summary of the parameters for the determined processing windows for the true strain of 1 according to the approach of Prasad and Murty.

The analysis of the processing maps in accordance with Prasad’s and Murty’s criterion makes it possible to ascertain that Murty’s criterion presents deformation mechanisms set against energy-focused ways of seeing the process in a more comprehensive way, in particular, within the range of changes in the course, and in the values of the efficiency of the power dissipation parameter *η*%, and also in that of the material structure. The differences between the predicted results for the processing windows and the location of them are small, and the precision of predictions in accordance with Murty’s criterion is more stable, and, ipso facto, superior. Murty’s criterion presents the regimes of process instability in accordance with the equation (7) more broadly, because it includes the values of the efficiency of the power dissipation parameter *η* and the strain-rate-sensitivity parameter m for verifying the metallurgical process instability. Upon the basis of the processing maps for the investigated steels (Figure 3), it is possible to observe that the amount of deformation exerts a significant influence upon identifying the processing windows and the area of instability. Processing maps provide valuable technological information for designing the bulk metal working process, such as the forging of drop forgings having diversified surfaces (in terms of cavities, ribs or splines), and the degree of the difficulty of processing. That is relevant to, in particular, the assessment of the intensiveness of the material forming process and in determining the level of the influence of the set of intermediate strains for achieving the correct die impression, filling and obtaining the presumed shape of a good quality forged part. Taking advantage of the method of intermediate stages may constitute a significant cognitive contribution to the assessment of the material forming design correctness.

**Figure 4:**
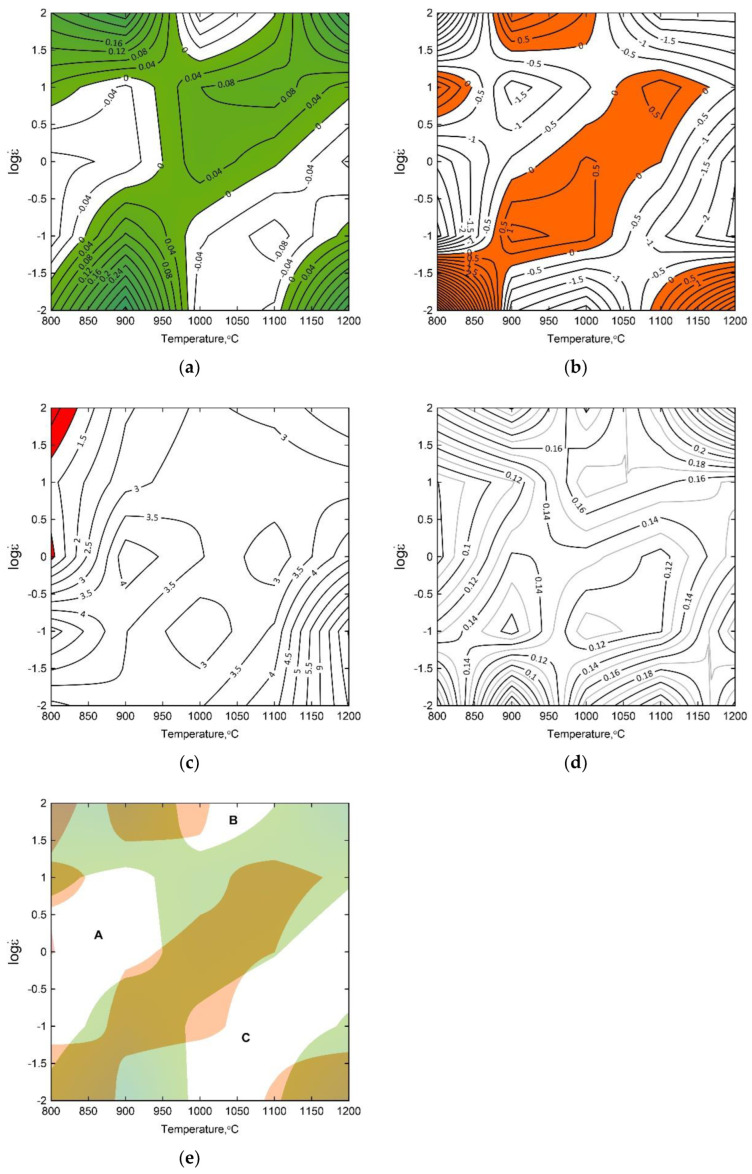
Contour map for the *η*_criterion_ (**a**), contour map for the *s*_criterion_ (**b**), contour map for the temperature-sensitivity parameter of flow stress s (**c**), the maps of changes in the strain-rate-sensitivity parameter *m* (**d**) and the processing map based upon Gegel’s stability criterion (**e**) for the 4130 steel for the true strain of 1, A, B, C-domains of flow stability.

The complex maps, presented in Figure 4, were developed as the result of superimposing four inequalities describing the stability criteria in accordance with Gegel’s approach for the true strain of 1. The map shows the stable domains (white areas) and the instability regimes (colored). Three stability areas, marked as A, B and C, having the most advantageous combination of the parameters *m*, *η*_criterion_, *s* and *s*_criterion_ were identified for the 4130 steel.

### 3.3. Processing Maps Combined with the Analysis of the Microstructure of the Investigated Steel

The analysis of the microstructures of the samples deformed in the compression on the Gleeble 3800 simulator was performed. The microstructure of the investigated steel developed as the result of intense cooling after deformation in the austenite region. The applied cooling resulted in a diffusion-free transformation of the austenite into martensite or in intermediate transformation into bainitic structures. 

**Figure 5:**
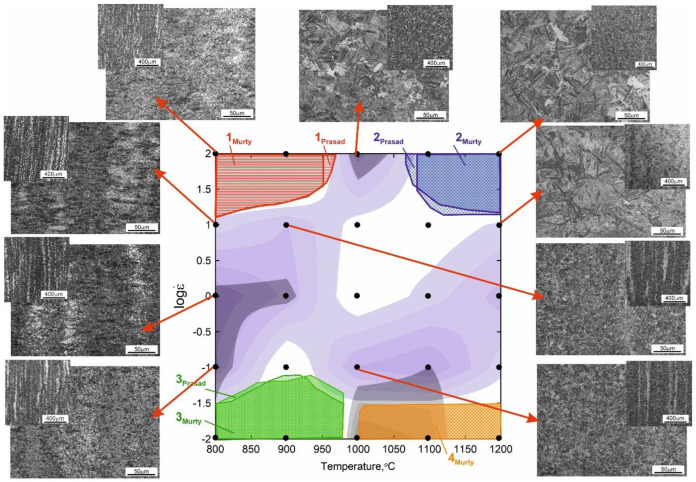
Complex processing map combining all the analyzed flow–stability criteria correlated with characteristic microstructural changes observed in the deformed samples. The areas in gray shades correspond to the material flow instability parameters. White areas correspond to processing parameters leading to stable material flow.

It can be observed that increasing the deformation temperature and strain rate favor the homogenization of the tested steel, causing the banded structure to disappear (Figure 5). However, even at higher strain rates at a deformation temperature of 1200 °C, banding in the microstructure of the samples can still be noticed. This proves the important role of temperature and annealing time for the austenitic range in the diffusion of alloying elements and carbon, favoring the homogenization of the chemical composition of austenite. Figure 5 presents the complex processing map combining all the analyzed flow–stability criteria correlated with the characteristic microstructural changes observed in the deformed samples. The left-hand upper corner of the processing map has the parameter value of s < 1, and therefore, the temperature and microstructure instability of the material. The right-hand upper corner of the map (strain rate increasing up to 100 s^–1^) indicates an increase in material strength resulting from work hardening. The left-hand bottom corner of the complex processing map indicates the deformation heterogeneity, often connected with transition bands between two parts of austenite grains (banding). The right-hand bottom corner of the map is characterized by microstructural instability connected with the growth of the austenite grains in the matrix of a deformed material.

The banding visible in the microstructure of the deformed compression samples is the result of microsegregation of the chemical composition (mainly phosphorus and manganese). In the normalized (initial) state, it is faintly visible; however, accelerated cooling from the austenite range (austenitic structure was deformed) resulted in a bainitic transformation, which made again the areas of chemical composition segregation visible due to the formation of the different bainite morphologies as a result of the different hardening of micro-areas of the different chemical composition.

### 3.4. Industrial Forging Test and the Analysis of the Microstructure of Forged Part

**Figure 6:**
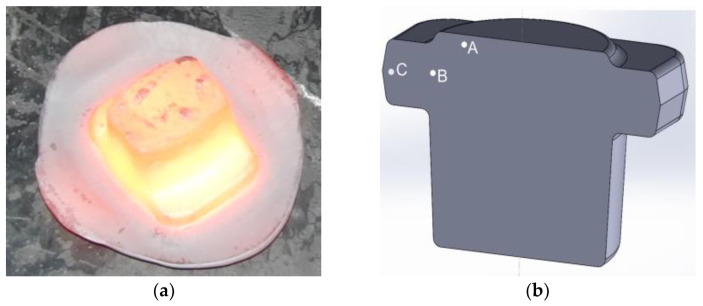
The flange forged on hammer (**a**), the flange model with areas of metallographic investigations marked (**b**). A, B, C – areas of metallographic observations on the forged part cross-section.

A comprehensive approach to the issue of die forging enables a multi-criteria analysis of the thermomechanical process of forging complex-shaped forgings—the typical representative of which is the flange-type forging—according to the parameters determined on the basis of the processing maps ensuring the desired microstructure and mechanical properties. The forging of 4130 steel in industrial conditions was carried out on a double-action hammer in accordance with the established parameters of the forming process related to the processing window 2 according to Prasad’s and Murty’s criteria. The billet was heated up to the temperature of 1200 °C in an electrical furnace and forged in two operations: initial upset forging and forging in a finishing impression. After the forging process, the forged part was cooled with forced air. A flange forged on hammer is presented in Figure 6a. The samples for the metallographic investigations were taken from the cross-section of the forged part (Figure 6b).

**Figure 7:**
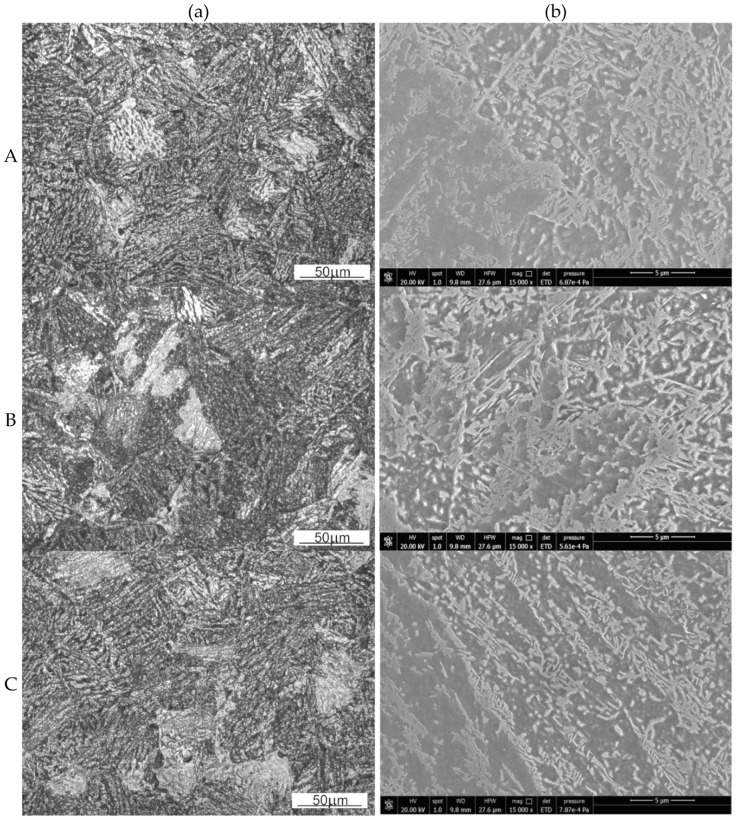
The microstructure of the 4130-steel flange in pre-selected points of the microstructure observations (**A**–**C**): (**a**) light microscope, (**b**) scanning microscope.

Figure 7 shows selected optical (a) and scanning electron micrographs (b) at the points selected for the analysis (see Figure 6b) of the microstructure of the flange forging. The mean primary austenite grain sizes were calculated using Jeffries method. The microstructure has the largest grains in area B (2956 μm^2^), smaller in area A (2888 μm^2^), and the smallest in area C (1769 μm^2^). This corresponds to the areas of increasing strain in the forged part. The characteristic features of the pearlitic structure indicate its more intense supercooling in the areas of more intense cooling of the forged part, where the microstructure has the morphology of the upper bainite. The microstructure indicates the least intense cooling at area B and the most intense cooling at areas A and C.

The transformation of austenite to fine pearlite (fine-plate structure) is more diffusive in nature than the transformation of austenite to upper bainite. The diffusive nature of these transformations (in the case of pearlite nucleating mainly at austenite grain boundaries) makes it difficult to reconstruct the austenitic structure formed in this case by the plastic deformation of austenite and the processes of its recovery and dynamic recrystallization. However, these transformations are often similar in temperature range (always, however, perlite is formed at higher temperatures than bainite), which often results, as in the present case, in the absence of a clear distinction between the areas of fine perlite and upper bainite. Increasing the cooling rate facilitates a less diffusive transformation (in this case, resulting in formation of upper bainite) and limits the extent to which a more diffusive pearlitic transformation occurs. However, the austenite grain refinement (area C in Figure 6b), introducing more high-energy grain boundaries, promotes the diffusion that is facilitated along these boundaries. This leads to reducing the hardenability, which favors the occurrence of pearlitic transformation and therefore a mainly diffusive transformation. Therefore, considering area C shown in Figure 6b, two opposite phenomena (accelerated cooling and grain refinement) result in a similar character of the morphology of cementite precipitates (Figure 7b) at this area of the forged part cross-section as well as at areas B and C.

## 4. Conclusions

The main conclusions of this study can be summarized as follows:

- The conducted assessment of process stability and instability in accordance with Prasad’s and Murty’s criterion for the true strain of 1 showed three processing windows developed in accordance with Prasad’s criterion and four processing windows developed in accordance with Murty’s criterion.
- Upon the basis of the processing maps of the investigated steel, it is possible to observe that the level of strain has a significant influence on identifying the processing windows and the area of instability.
- The processing map developed according to Gegel’s approach, as compared to the processing maps obtained in accordance with Prasad’s and Murty’s criteria, showed three stability areas. It is, however, difficult to clearly identify the most favorable combinations of the processing parameters based on them. This map should be treated as general support for determining thermomechanical processing parameters.
- Processing parameters presented in processing window 2, developed according to Prasad’s and Murty’s criteria, are recommended for designing the technology of forging of the investigated steel. Industrial forging tests should be carried out under the following thermomechanical conditions: T=1075–1200 °C and $\dot{\varepsilon }=15.8–100{\;s}^{–1}$ on hammers or other high-strain-rate forging machines. Such parameters ensure the homogeneity and stability of the material flow in a forged part, which was confirmed by the successful forging of 4130 steel in industrial conditions.

## Figures and Tables

**Table 1:** The chemical composition of the investigated steel.

	Chemical Composition, wt. %											
Fe	C	Mn	Ni	Cr	P	Si	Mo	S	V	Sn	Al	N
Bal.	0.29	0.55	0.11	1.10	0.007	0.17	0.22	0.012	0.004	0.007	0.027	0.008

**Table 2:** Suggested thermomechanical processing parameters for the investigated steel, determined in processing windows for a true strain of 1 according to Prasad’s and Murty’s criterion.

Window		Prasad’s Approach	Murty’s Approach
1	The efficiency of power dissipation	$\eta_{P}=18–36\%$	$\eta_{M}=18–32\%$
	The range of temperatures	T=800–975 °C	T=800–950 °C
	The range of strain rate	$\dot{\varepsilon }=12.5–100{\;s}^{–1}$	
2	The efficiency of power dissipation	$\eta_{P}=30–46\%$	$\eta_{M}=32–44\%$
	The range of temperatures	T=1075–1200 °C	
	The range of strain rate	$\dot{\varepsilon }=15.8–100{\;s}^{–1}$	
3	The efficiency of power dissipation	$\eta_{P}=10–24\%$	$\eta_{M}=10–30\%$
	The range of temperatures	T=800–980 °C	
	The range of strain rate	$\;\dot{\varepsilon }=0.01–0.06{\;s}^{–1}$	
4	The efficiency of power dissipation	-	$\eta_{M}=28–38\%$
	The range of temperatures	-	T=1000–1200 °C
	The range of strain rate	-	$\dot{\varepsilon }=0.01–0.03{\;s}^{–1}$

## Data Availability

Not applicable.
